# Gegen Qinlian Decoction Coordinately Regulates PPARγ and PPARα to Improve Glucose and Lipid Homeostasis in Diabetic Rats and Insulin Resistance 3T3-L1 Adipocytes

**DOI:** 10.3389/fphar.2020.00811

**Published:** 2020-06-11

**Authors:** Jun Tu, Shuilan Zhu, Bingtao Li, Guoliang Xu, Xinxin Luo, Li Jiang, Xiaojun Yan, Ruiping Zhang, Chen Chen

**Affiliations:** ^1^Research Center for Differentiation and Development of TCM Basic Theory & Jiangxi Province Key Laboratory of TCM Etiopathogenesis, Jiangxi University of Traditional Chinese Medicine, Nanchang, China; ^2^Key Laboratory of Pharmacology of Traditional Chinese Medicine in Jiangxi, Jiangxi University of Traditional Chinese Medicine, Nanchang, China; ^3^Institute of Materia Medica, Chinese Academy of Medical Sciences and Peking Union Medical College, Beijing, China; ^4^Endocrinology and Metabolism, SBMS, Faculty of Medicine, University of Queensland, Brisbane, QLD, Australia

**Keywords:** PPARγ, PPARα, lipid metabolism, glucose homeostasis, Gegen Qinlian Decoction

## Abstract

Gegen Qinlian Decoction (GQD), a well-documented traditional Chinese Medicine (TCM) formula, was reported with convincing anti-diabetic effects in clinical practice. However, the precise antidiabetic mechanism of GQD remains unknown. In this study, the anti-hyperglycemic and/or lipid lowering effects of GQD were demonstrated in high-fat diet with a low dose of streptozotocin induced diabetic Sprague-Dawley rats and insulin resistance (IR)-3T3-L1 adipocytes. GQD treatment increased expression and activity levels of both PPARγ and PPARα in adipocytes, which transcriptionally affected an ensemble of glucose and lipid metabolic genes *in vivo* and *in vitro*. The results clearly indicated that GQD treatment intervened with multiple pathways controlled by concomitantly downstream effects of adipocytic PPARγ and PPARα, to influence two opposite lipid pathways: fatty acid oxidation and lipid synthesis. Antagonist GW9662 decreased the mRNA expression of *Pparγ* and target genes *Adpn* and *Glut4* whereas GW6471 decreased the mRNA expression of *Pparα* and target genes *Cpt-1α*, *Lpl*, *Mcad*, *Lcad*, *Acox1, etc*. Nuclear location and activity experiments showed that more PPARγ and PPARα shuttled into nuclear to increase its binding activities with target genes. GQD decreased the phosphorylation level of ERK1/2 and/or CDK5 to elevate PPARγ and PPARα activities in IR-3T3-L1 adipocytes through post-translational modification. The increase in p-p38MAPK and SIRT1 under GQD treatment may be attributed to partially reduce PPARγ adipogenesis activity and/or activate PPARα activity. Compared with the rosiglitazone-treated group, GQD elevated *Cpt-1α* expression, decreased diabetic biomarker *Fabp4* expression, which produced an encouraging lipid profile with triglyceride decrease partially from combined effects on upregulated adipocytic PPARγ and PPARα activities. These results suggested that GQD improved diabetes by intervening a diverse array of PPARγ and PPARα upstream and downstream signaling transduction cascades, which jointly optimized the expression of target gene profiles to promote fatty acid oxidation and accelerate glucose uptake and utilization than PPARγ full agonist rosiglitazone without stimulating PPARα activity. Thus, GQD showed anti-diabetic/or antihyperglycemic effects, partially through regulating adipocytic PPARα and PPARγ signaling systems to maintaining balanced glucose and lipid metabolisms. This study provides a new insight into the anti-diabetic effect of GQD as a PPARα/γ dual agonist to accelerate the clinical use.

## Introduction

Diabetes mellitus (DM) is characterized by high blood glucose levels due to relatively deficiency in both insulin action and secretion. Epidemiological survey analysis showed that the prevalence of DM had increased significantly in recent decades, affected more than 114 million patients in 2010 China (Xu et al., 2013). Insulin resistance (IR), an early detectable pathological defect in type 2 DM (T2DM), is the predominant factor causing diabetes (> 90%). T2DM correlates with peripheral IR in adipose, liver, and skeletal muscle tissues. Adipocyte peroxisome proliferator activated receptor-gamma (PPARγ) is the molecular target of thiazolidinediones (TZDs)-based drugs, regulating the transcription level of insulin-responsive genes to enhance insulin sensitivity in peripheral tissues in T2DM (Ahmadian et al., 2013). However, TZDs-based anti-diabetic drugs have various adverse effects such as fluid retention, adipogenic weight gain, and cardiac failure, mainly due to the high potency of TZD drug as a strong PPARγ stimulator. Considering that PPARα and PPARγ play complementary roles to each other in the regulation of lipid homeostasis by modifying lipid transport, storage, and metabolism. PPARα/γ dual agonists overcomes the undesirable side effects of sole PPARγ agonist in treating T2DM.

Gegen Qinlian Decoction (GQD), a well-documented traditional TCM formula, was used for treating diarrhea and dysentery for a long time. Recently, GQD was effective and safe in glycemic control with increased insulin sensitivity for patients in a randomized double blind placebo controlled clinical trial and a 5-year retrospective study (Xu et al., 2015; Tian et al., 2016). However, the precise anti-diabetic mechanism of GQD remains unknown.

Recently, anti-diabetic mechanism studies of GQD mainly focused on liver and skeletal muscle IR, our study intended to explore the molecular mechanism of GQD in regulating adipocytic glucose and lipid metabolism. Our preliminary research showed anti-hyperglycemic effect of GQD through stimulation of adipocytic *Pparγ* expression to improve insulin sensitivity and glucose transport *in vivo* and *in vitro* (Luo et al., 2017). In this study, GQD showed obvious lipid-lowering effects, especially reduction of total triglycerides (TGs). We hypothesized that GQD was a non-TZD substitute as a PPARγ/α dual activators in the white adipose tissue (WAT) of diabetic rats and/or IR3T3-L1 adipocytes for anti-hyperglycemic effect. It aimed to provide an insight view of the antidiabetic mechanisms of GQD to accelerate the clinical use.

## Materials and Methods

### Chemicals and Reagents

Gegen [*Puerariae lobata* (Willd.) Ohwi, batch number: 0912014], Huangqin (*Scutellariae balcalensis* Georgi, batch number: 0911002), Huanglian (*Coptidis rhizoma* Franch., batch number: 0905024), and Zhigancao (*Glycyrrhizae uralensis* Fisch., batch number: 0911018) were purchased from the Beijing Shuangqiao Yanjing Herbal Pieces Factory and were identified by Professor Yi Rao, National Pharmaceutical Engineering Center for Solid Preparation in Chinese Medicine, Jiangxi University of Traditional Chinese Medicine, China. A list of the full taxonomic names of all species used have been included in **Table S1**. The standard chemical samples from four herbs were purchased from Sichuan Victory Biological Technology Co. Ltd. Streptozotocin (STZ), 3-isobutyl-1-methylxanthine (IBMX), dexamethasone (DEX), insulin, metformin, and rosiglitazone were obtained from Sigma-Aldrich (St. Louis, MO, USA).

β-Actin, β-tubulin, PPARγ, SIRT1, ACC1, FASn, p38MAPK, p-p38MAPK, ERK1/2, and p-ERK1/2 primary antibodies were purchased from CST (Danvers, USA, Cat No: 4970, 2128, 2435, 9475, 3676, 3180, 8690, 4511, 4695, and 4370); PPARα, p-PPARα (S12), p-PPARγ (S112), and Lamin B1 primary antibodies were obtained from Abcam (Cambridge, UK, Cat No: ab8934, ab3484, ab195925, and ab133741); PGC-1α primary antibody and goat anti-Rabbit IgG (H+L) secondary antibody were purchased from Invitrogen (Carlsbad, USA, Cat No: PA5-38201 and 31460). CDK5 and SREBP-1C primary antibodies were purchased from Cusabio Biotech (Houston, USA, Cat No: G0117A and E1123A).

### Analysis of the Major Components of Each Single Herb by High Performance Liquid Chromatography (HPLC)-UV Method

Each single herb of GQD prescription was analyzed by a HPLC-UV method described in Wagner's study with minor modification relating to the instrument and chromatographic conditions (Wagner et al., 2011). A chromatographic column (Shim-pack XR-ODS III, 2.0 mm × 7.5 mm; 1.6 μm, Shimadzu HPLC packed column) was used to perform chromatographic separation at 35°C. The mobile phase was acetonitrile-0.05% phosphoric acid in gradient elution. By referring to the standards of the Chinese Pharmacopoeia (2010 edition), the chemical identification of the main compounds of each herb were as showed in **Supplementary File** (**Figure S1**).

### Preparation of GQD Decoction and GQD-Containing Serum (GQD-CS) Analysis of the Major Components of GQD by HPLC-UV Method

GQD are generally prepared by extracting dried medicine herbs with boiling water, then administrated as the water extracts named decoction. The composing ratio of the four herbs of GQD formula including Gegen, Huangqin, Huanglian and Zhigancao was recorded as “8:3:3:2” as a well-known classical decoction in classic book “Shanghan Lun” about 1900 years ago. The four dried herbs were immersed in eight times the amount of distilled water (v/w) for 30 min and extracted twice: once for 1 h and then 40 min for the second time using a decoction pot. The supernatants were pooled together and concentrated to 1 g of crude drug per milliliter (drug extract ratio; 1:1) by a rotary evaporator. The GQD concentrates (1 g/ml) were finally stored at 4°C for the subsequent studies.

The GQD-CS were prepared and detected according to the same method described previously by our research group (Zhang et al., 2016). Briefly, male SD rats (250–280 g) were obtained from Beijing Charles River Laboratories and divided into three groups. In the first and second groups, rats were orally administered GQD for 11.55 ml/kg daily and the blood was obtained by cardiac puncture 1 h after the last administration and designated as the GQD-CS respectively. In the third group, the rats were orally administered normal saline and the serum was collected as the control.

Both serum samples were inactivated by heating to 56°C for 30 min, then filtered and stored at −20°C until measurements.

### Analysis of the Major Components of GQD and GQD-CS by UPLC-MS/MS Method

For the very low concentrations of some components in the GQD-CS, it was not possible to apply the HPLC method for its quality control. Thus, the ultra-high performance liquid chromatography-mass spectrometry (UPLC-MS/MS) method with triple quadrupole mass spectrometer ABQ-TRAP5500 was used to perform the quality control of GQD-CS and GQD. In brief, GQD-CS was extracted with methanol and acetonitrile, after which the rest of the steps were essentially the same process described previously by our research group (Zhang et al., 2016). The chemical identification of the main compounds of GQD and GQD-CS were identified.

### Animal Model and Drug Administration

Eight-week-old male Sprague-Dawley (SD) rats with body weight of 180–200 g were purchased from Hunan Slac Jynda Laboratory Animal Company (Hunan, China) and maintained at 23 ± 2°C with a relative humidity of 55 ± 10%, and under a 12 h light-dark cycle in experimental animal facilities. The rats were allowed *ad libitum* access to food and sterilized water. Before the experiment, all rats were adaptively fed with chow diet (Shanghai Slac Laboratory Animal Company, Shanghai, China) including 5% fat, 23% protein, and 53% carbohydrate for 1 week. Eight SD rats were randomly selected as normal control group with chow diet until the ending of this animal study. The remaining SD rats were fed a high-fat diet (HFD) for four consecutive weeks. The HFD (D#12492) was purchased from Research Diets Company (New Brunswick, USA) including 60% fat, 20% protein, and 20% carbohydrate. Subsequently, the diabetic rat model was developed with a modified method of our group (Zhang et al., 2013). Briefly, SD rats were injected by tail vein with a small dose of STZ (30 mg/kg^−1^, dissolved in 0.1 M sodium citrate buffer, pH 4.4) after 12 h fast. One week later, blood samples were collected by tail cutting, then fasting blood glucose was measured by blood sugar meter with strips (Roche, Mannheim, German).

Diabetic rats with a fasting blood glucose of ≥16.7 mmol/L and the control rats were fed with chow diet, then randomly divided into four groups: (1) control group (control rats treated with saline in a matched volume); (2) diabetic model group (diabetic rats treated with saline in a matched volume); (3) diabetic metformin-treated group (diabetic rats were treated with metformin at 0.2 g/kg); (4) diabetic GQD-treated group (diabetic rats were treated with middle dose of GQD at 11.55ml/kg). GQD, metformin or saline were administered *via* oral gavage twice a day for 13 weeks. Dose-effect relationship curves of GQD showed that the middle dosage had the highest efficacy in the same diabetic SD rat model (Huang et al., 2017), it is consistent with our previous animal study.

The use of animals was approved by the animal ethics committee of Jiangxi University of Traditional Chinese Medicine (20140301). All animal experiments were carried out in a manner consistent with the “Regulations on the Management of Laboratory Animals” promulgated by the State Science and Technology Commission (2013 Version).

### Body Weight and Serum Biochemical Analysis

The body weight and fasting blood glucose (FBG) were measured weekly during 13 weeks GQD treatment period. At the end of the experiment, 12 h fasting rats were anesthetized by phenobarbital sodium (150 mg/kg), rat blood samples were collected from the portal vein into precooled tubes containing EDTA and centrifuged at 5,000 rpm for 15 min at 4°C to isolate serum. Serum TG, total cholesterol (TC), low density lipoprotein cholesterol (LDL-C), and high-density lipoprotein cholesterol (HDL-C) were measured using commercial enzymatic assay kits (Jiancheng Bioengineering, Nanjing, China). WAT from epididymal area was collected and immediately frozen at −80°C for further analysis.

### Cell Culture and Induction of IR-3T3-L1 Adipocytes Model

The 3T3-L1 mouse preadipocyte cell line was obtain from the American Type Culture Collection (ATCC, Manassa, USA, Cat No; CL-173, Batch No: 62996847). The 3T3-L1 preadipocytes were grown in high-glucose DMEM containing 10% new-born calf serum and differentiated into adipocytes with 0.5 mM IBMX, 1 μM DEX, and 10 μg/ml insulin in culture medium (Sangeetha et al., 2013). More than 90% of 3T3-L1 preadipocytes were differentiated to mature adipocytes in 8–12 d.

IR-3T3-L1 adipocytes model was induced by 1 μM DEX in 25 mM high glucose DMEM medium with 10% fetal bovine serum (FBS) for 96 h described previously by our research group (Luo et al., 2017). The glucose content in the culture medium was quantified by GOD-POD method using glucose assay kit (Jiancheng Bioengineering, Nanjing, China). Compared with the normal group, calculated glucose consumption (GC) of the DEX-treated group showed a significant decrease, which suggested that IR model of 3T3-L1 adipocytes was built successfully.

### Subgrouping and Administration of IR-3T3-L1 Adipocytes

The 3T3-L1 adipocytes were randomly divided into six groups in serum-free 25 mM high-glucose DMEM with 5% FBS and 1 μM DEX for the time and dose-effect experiment at different time points (6, 12, 24, 30, 36, 48, 54 h): (1) control group + 15% control rat serum; (2) IR model group + 15% rat control serum; (3) 10 μM rosiglitazone group + 15% control rat serum; (4) 5% GQD-CS/5% Gegen-CS-treated group + 10% control rat serum; (5) 10% GQD-CS/10% Gegen-CS-treated group + 5% control rat serum; and (6) 15% GQD-CS/15% Gegen-CS-treated group. All groups were supplemented to 15% rat serum. Afterward, glucose content was quantified by glucose assay kits and cell viability by cell counting kit-8 (CCK8 kit, Dojindo Molecular Technologies Inc, Japan). Cell viability = IR group *A*_450/_control group *A*_450_× 100%. After 24 h administration, ADPN content in the culture medium was quantified by Mouse ADPN ELISA Kit (Invitrogen, Carlsbad, USA). The cells were rinsed with PBS twice, then, stained by oil red O staining described in Sangeetha's study (Sangeetha et al., 2013).

### Detection of mRNA Expression

Total RNAs in rat WAT and 3T3-L1 adipocytes were separately isolated using RNeasy Lipid Tissue Mini Kit and RNeasy Mini Kit (Qiagen, Dusseldorf, Germany), then were reverse-transcribed to cDNAs using GoScript™ Reverse Transcription System (Promega, Madison, USA). In generally, adipocytic gene expression levels were detected by quantitative PCR (qPCR) on an ABI PRISM 7500 instrument or Bio-Rad CFX96 Touch instrument using the Power SYBR Green PCR Master Mix (Life Technologies, Carlsbad, USA). qPCR was performed using the following protocol: 1 cycle at 50°C for 2 min and 95°C for 10 min, followed by 40 cycles at 95°C for 15 s, 58–63°C for 30 s, and 68°C for 30 s. These sequences of rat and mouse qPCR primers and relative reactive conditions with annealing temperature were given in **Tables S2** and **S3**. The primers were synthesized by Sangon Biotech (Shanghai, China). All qPCR data were normalized to *β-actin* gene expression.

### Detection of Protein Expression and Activity

Total protein in rat WAT and 3T3-L1 adipocytes were separately isolated using Minute™ Total Protein Extraction Kit for Adipose Tissues/Cultured Adipocytes (Invent Biotechnologies, Plymouth, USA). Nuclear protein in 3T3-L1 adipocytes were isolated using NE-PER™ nuclear and cytoplasmic extraction reagents with Halt™ protease and phosphatase inhibitor cocktail. Fifteen to 20 μg total protein samples or 8–10 μg nuclear protein samples were separated by SDS-PAGE and electro-transferred onto PVDF membranes (Millipore, Billerica, USA). The membranes were incubated in blocking buffer with 5% fat-free milk/5% BSA at 4°C for 1 h; incubated with primary antibodies such as β-actin (1:1,000), β-tubulin (1:1,000), PPARγ/α (1:1,000), p-PPARγ (Ser112)/p-PPARα (Ser12) (1:500), p38MAPK (1:1,000), p-p38MAPK (1:1,000), ERK1/2 (1:1,000), p-ERK1/2 (1:2,000), CDK5 (1:1,000), SIRT1 (1:1,000), and Lamin B primary antibodies (1:1,000) overnight at 4°C; then washed with TBST for three times; and eventually incubated with horseradish peroxidase-conjugated anti-rabbit antibodies for 1 h, washed with TBST for three times and developed with Clarity™ Western ECL Substrate Kit

(Bio-Rad, Hercules, USA) or SuperSignal™ West Pico Rabbit IgG Detection Kit (Pierce, Rockford, USA). Visualized chemiluminescence signals were detected using Molecular Imager (ChemiDOC™ XRS+) with Image Lab™ Software (Bio-Rad, Hercules, USA). The target protein expression levels were normalized to that of β-actin or β-tubulin expression levels. The nuclear protein expression levels were normalized to that of Lamin B expression level. The activation of PPARγ and PPARα was detected by PPARγ and PPARα transcription factor assay kits (Abcam, Cambridge, UK), separately.

### PPARγ/α Antagonist Experiment

In the PPARγ/PPARα antagonist experiment, the IR-3T3-L1 adipocytes were divided into six groups with a 24 h treatment in high-glucose DMEM with 5% FBS and 1 μM DEX: (1) IR model group + 15% control rat serum + 0.062% DMSO; (2) IR model group + 15% control rat serum + 10 μM GW9662/GW6471; (3) 10 μM rosiglitazone group + 15% control rat serum + 0.062% DMSO; (4) 10 μM rosiglitazone group + 15% control rat serum + 10 μM GW9662; (5) 10% GQD-CS-treated group + 5% control rat serum + 0.062% DMSO; (6) 10% GQD-CS-treated group + 5% control rat serum + 10 μM GW9662/GW6471. The mRNA expression levels of PPARγ and PPARα with downstream regulatory genes were detected using the qPCR method as shown in above.

### Statistics Analysis

Statistical analyses were performed using GradphPad Prism 6 software (La Jolla, CA, USA). All results were presented as the mean ± SEM. Statistical analysis was performed *via* analysis of variance (one-way ANOVA for column analyses or two-way ANOVA for grouped analyses followed by Dunnett's multiple comparisons test) followed by the Student-Dunnet test of significance, 251 whereas *t*-test was used to compare two groups. Differences were considered statistically significant at *P* < 0.05.

## Results

### Anti-Hyperglycemic Effect and Lipid-Lowering Effects in the GQD-Treated Diabetic SD Rats

The major compounds, including puerarin, daidzein, wogonoside, baicalin, baicalein, jatrorrhizine, palmatine, berberine, and liquiritin, in GQD and GQD-CS were detected by UPLC MS/MS method respectively (**Table 1**).

**Table 1:** Contents of marker compounds in Gegen Qinlian Decoction-containing serum (GQD-CS) and GQD as determined by performance liquid chromatography tandem mass spectrometry (UPLC-MS/MS).

Herbs	Chemical	Peak time	Concentration (ng/ml)	
	name	(min)	GQD-CS	GQD
Kudzuvine root	Puerarin Daidzein	1.7 5.7	81.991 ± 8.603 522.326 ± 54.908	564.266 ± 29.624 28.237 ± 1.53
Baical skullcap root	Wogonoside Baicalin Baicalein	6.6 7.4 7.4	487.282 ± 30.802 28.148 ± 1.398 1.604 ± 0.112	577.588 ± 28.879 15.329 ± 0.613 1.171 ± 0.059
Golden thread	Jatrorrhizine Palmatine Berberine	3.6 4.5 4.6	0.004 ± 0.000 0.049 ± 0.003 0.069 ± 0.004	1.852 ± 0.074 537.471 ± 32.248 337.728 ± 23.641
Liquorice root	Liquiritin	3.2	5.777 ± 0.505	2.127 ± 0.149

GQD, Gegen Qinlian Decoction; GQD-CS, Gegen Qinlian Decoction-containing serum.

**Figure 1:**
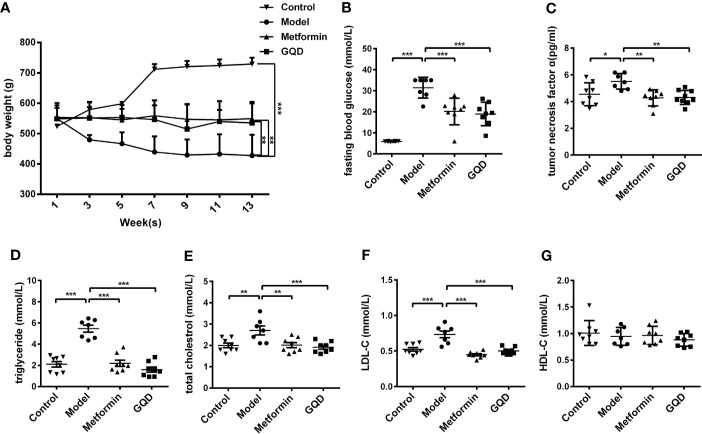
Gegen Qinlian Decoction (GQD) improved glucose and lipid metabolism without obvious weight change in diabetic Sprague-Dawley (SD) rats. **(A)** Time-effect of body weight was measured weekly. GQD treatment for 13 weeks, body weight values were expressed as the mean ± SEM, n=8 per group except n=7 for diabetic group since 11^th^ week. ^**^P < 0.01 and ^***^P < 0.001 when compared with the diabetic group at 13^th^ week. **(B)** Fasting blood glucose (FBG); **(C)** Serum tumor necrosis factor-α (TNF-α); **(D–G)** Serum lipid profiles including triglyceride, total cholesterol, low density lipoprotein cholesterol (LDL-C), and high density lipoprotein cholesterol (LDL-C) (HDL-C) were measured. All above values were expressed as the mean ± SEM, n=8 per group except n=7 for diabetic group. ^*^P < 0.05, ^**^P < 0.01, and ^***^P < 0.001 when compared with the diabetic group.

Compared with the diabetic group, body weight of GQD and metformin-treated rats was relatively stable after 6 weeks of treatments (**Figure 1A**). Moreover, FBG and tumor necrosis factor α (TNFα) was significantly decreased whereas the serum levels of TG, TC, and LDL-C were significantly decreased in GQD-treated diabetic SD rats after 13 weeks of drug administration (**Figures 1B–F**). However, the serum levels of HDL-C did not show any difference between different groups (**Figure 1G**).

### GQD Intervened PPARα and PPARγ Nodes Involved in Glucose and Lipid Metabolic Pathways in Diabetic Rats

**Figure 2:**
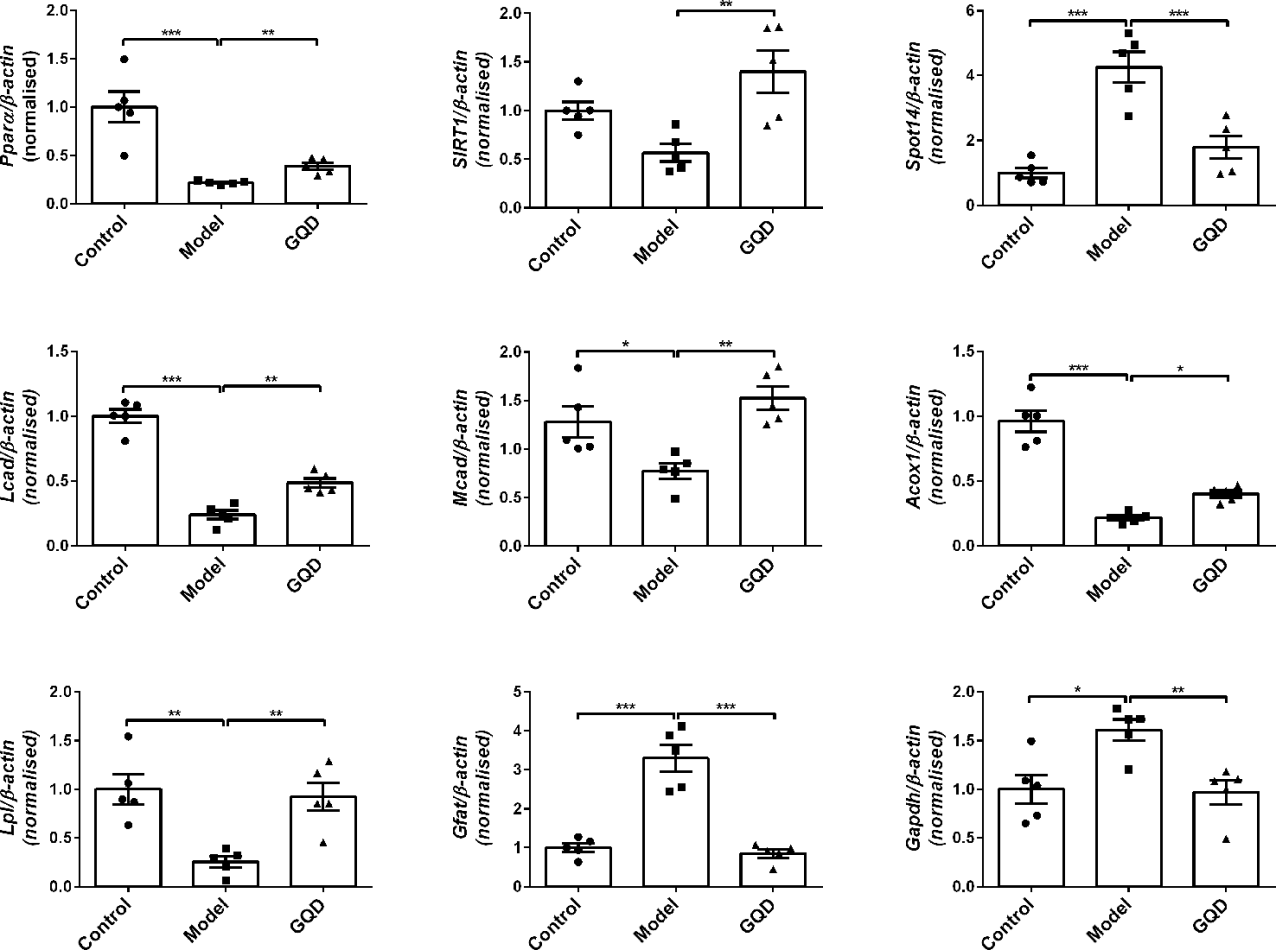
The mRNA expression levels of glucolipid metabolic genes including *Pparα, Acox1, Lcad, Mcad, Lpl, Sirt1, Srebp-1c, Spot14, Gfat*, and *Gadph* were detected by quantitative PCR (qPCR) method in rat white adipose tissue. Relative gene expression levels were corrected to the *β-actin* value. All values are expressed as the mean ± SEM, n=5 per group. ^*^P < 0.05, ^**^P < 0.01, and ^***^P < 0.001 when compared with diabetic group.

Compared with the diabetic rats, GQD-treated diabetic group showed not only significant increase in mRNA expression of PPARα but also elevation of transcriptional levels of downstream lipid metabolic genes *Lcad, Mcad, Acox1, Lpl*, etc. Moreover, the GQD-treated diabetic group showed significantly higher levels of mRNA expression of *Sirt1* but lower level of mRNA expression of *Spot14*, *Gfat*, and *Gadph* (**Figure 2**). Considering that *Gapdh* expression levels were not stable in different groups, relative gene expression levels were corrected to the *β-actin* value in this study.

**Figure 3:**
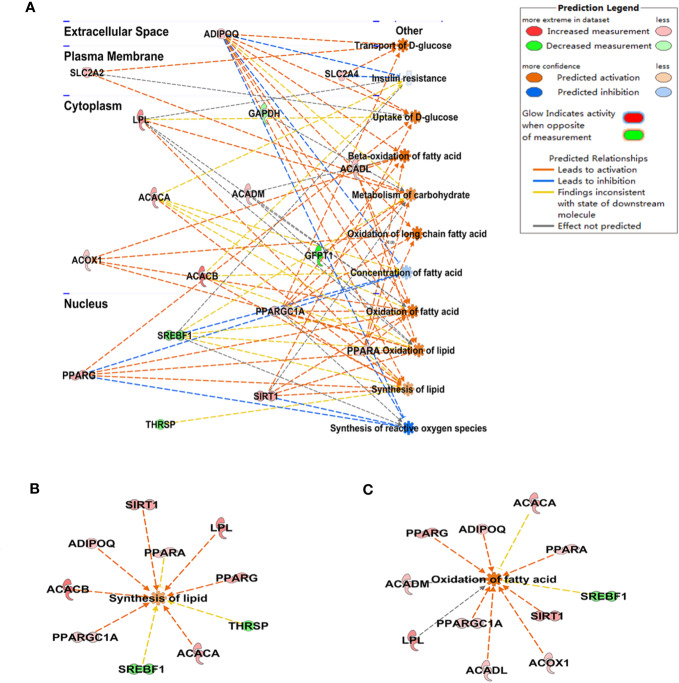
The schematic illustration of the intervened network with Gegen Qinlian Decoction (GQD) based on ingenuity pathway analysis (IPA). The disease-function pathway analysis with gene regulation network was shown in **(A)**; These significant expression changes of genes 518 involving in synthesis of lipid and the oxidation of fatty acid were shown in **(B, C)** and **Table S3**.

Based on the transcript data of the GQD-treated diabetic rats, a mapped ID coordinate regulatory network of glucose and lipid metabolism intervened by GQD with ingenuity pathway analysis (IPA) was illustrated in **Figure 3A** and **Supplementary File** (**Table S4**). Many important glucolipid metabolism pathways were upregulated including transport and uptake of D-glucose, β-oxidation of fatty acid, oxidation of fatty acids, metabolism of carbohydrate, oxidation and synthesis of lipid, etc. These signal molecules involving in IR pathway, concentration of fatty acid and synthesis of reactive oxygen species were downregulated. PPARγ and PPARα were therefore suggested as these important transcription factors involved in two opposite lipid pathways: fatty acid oxidation and lipid synthesis (**Figures 3B, C**) to recover adipocytic insulin sensitivity.

**Figure 4:**
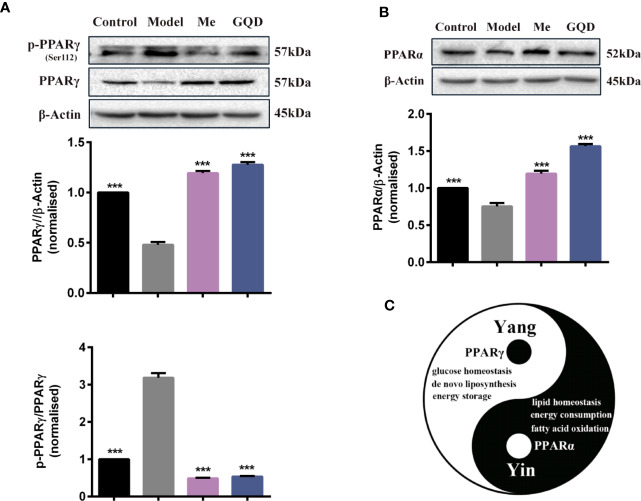
PPARγ/PPARα proteins with phosphorylated levels in rat white adipose tissue. **(A)** The expression levels of p-PPARγ (Ser112) and PPARγ; **(B)** The expression level of PPARα. **(C)** Prediction “Yin-Yang” working model of PPARγ and PPARα as complementary roles in the glucose and lipid metabolism. All values are expressed as the mean ± SEM, n = 3 per group. One-way ANOVA *with* Dunnett*'*s multiple comparisons tests were performed,^***^P < 0.001 when compared with diabetic group.

Compared with the diabetic group, protein levels of PPARγ and PPARα were significantly elevated in WAT of the GQD-treated diabetic rats (**Figure 4**). In addition, downregulated phosphorylated PPARγ (Ser112) was observed whereas phosphorylated PPARα (Ser12) was not detected due to relatively low expression. GQD might exert a better balanced PPARγ and PPARα action by increasing fatty acid oxidation and lipid synthesis as two opposite but related lipid metabolic pathways. This particular action was partially explainable according to “Yin and Yang” theory for both direction dynamic biological changes (Zhu, 2010) to produce beneficial effect. As a result, GQD improved overall glucose and lipid metabolism with both anti-hyperglycemic and lipid lowering effects.

### GQD-CS Increased Insulin Sensitivity in IR-3T3-L1 Adipocytes

**Figure 5:**
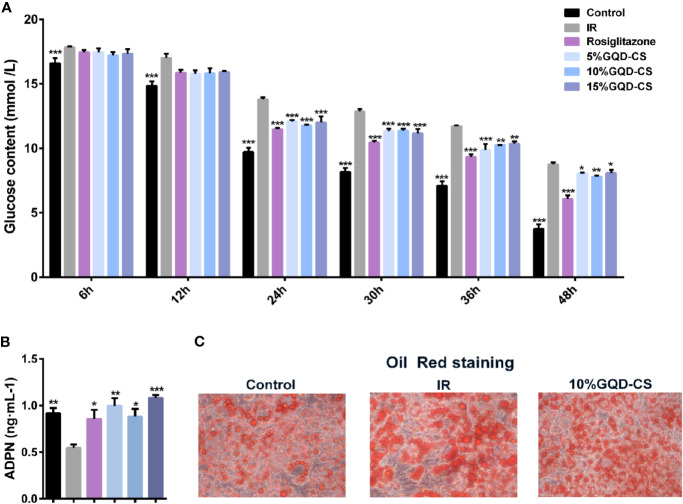
The anti-hyperglycemic with insulin sensitization effect of Gegen Qinlian Decoction-containing serum (GQD-CS) in the IR-3T3-L1 adipocytes. **(A)** Glucose contents in culture media; **(B)** ADPN content in culture medium. All values are expressed as the mean ± SEM, n = 5 per group. ^*^P < 0.05, ^**^P < 0.01, and ^***^P < 0.001 when compared with IR group. **(C)** Oil red staining (200X) for adipocyte morphology with intracellular adipose droplets.

All testing concentrations of GQD-CS significantly decreased glucose content in culture medium at 24, 30, 36, and 48 h treatment (**Figure 5A**). Moreover, GQD-CS increased adipokine ADPN level in culture medium at 24 h treatment in IR-3T3-L1 adipocytes (**Figure 5B**). Oil Red staining indicated that IR-3T3-L1 adipocytes had large amount of typical “ring-shape” lipid droplets whereas 10% GQD-CS treatment significantly decreased their number and size in IR-3T3-L1 adipocytes (**Figure 5C**).

### GQD-CS Regulated an Ensemble of Adipocytic Glucose and Lipid Metabolism Genes Through PPARγ and PPARα Nodes

**Figure 6:**
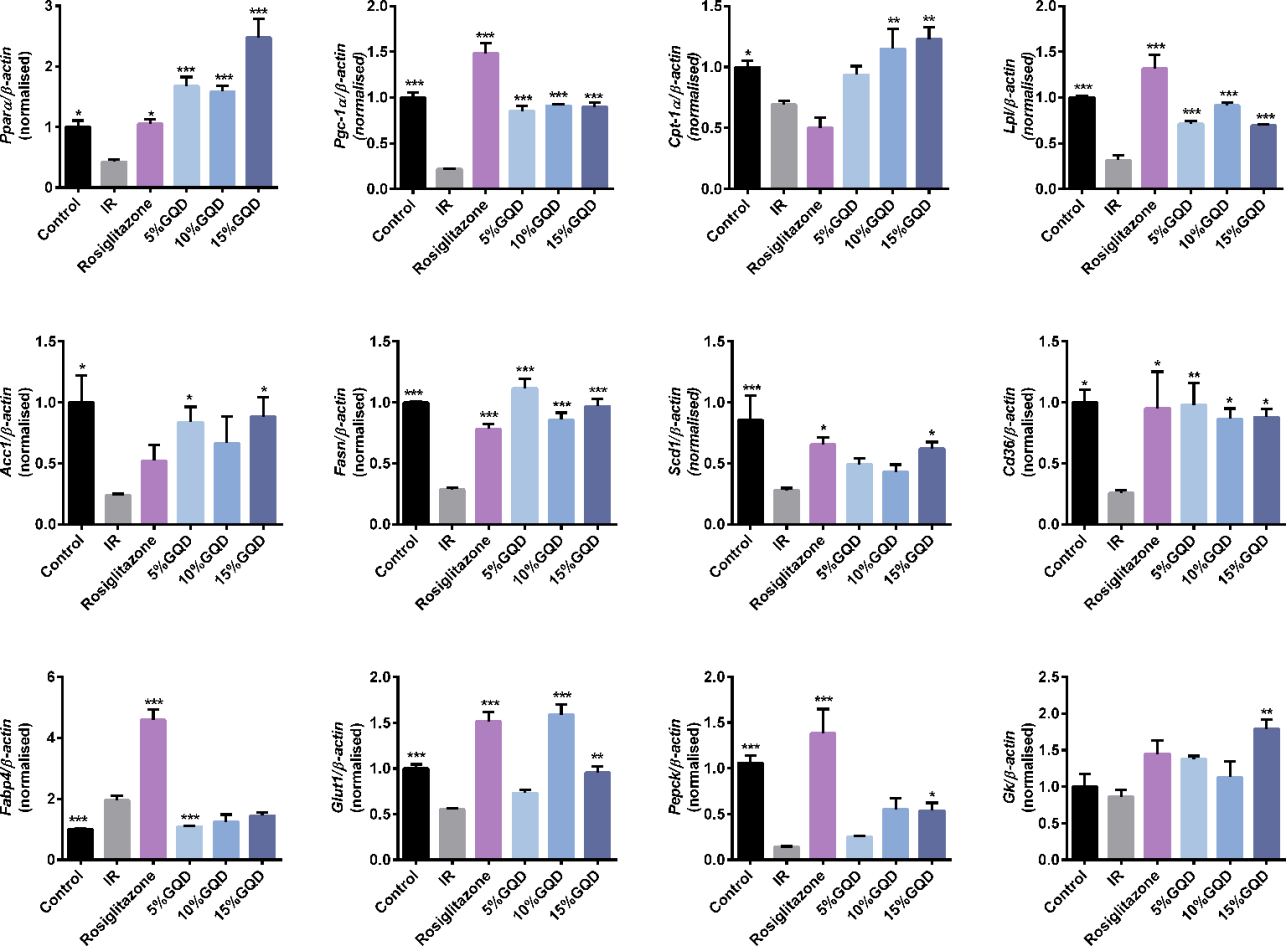
Gegen Qinlian Decoction-containing serum (GQD-CS) regulated an ensemble of adipocytic glucose and lipid metabolism genes in the IR-3T3- L1 adipocytes. All values are expressed as the mean ± SEM, n = 5 per group. ^*^P < 0.05, ^**^P < 0.01, and ^***^P < 0.001 when compared with IR group.

**Figure 7:**
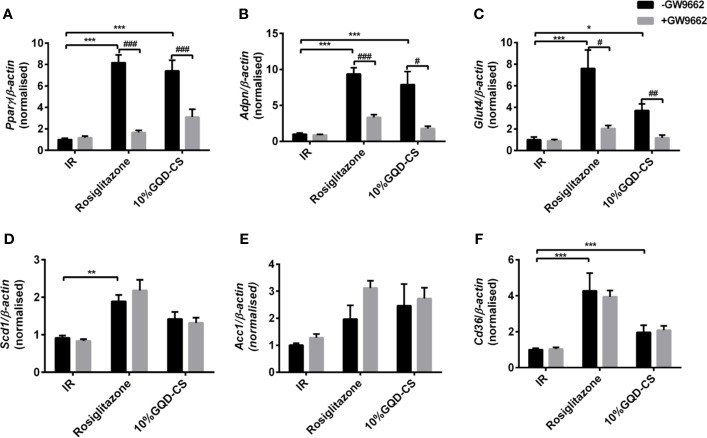
The mRNA expression level of glucolipid metabolism genes in presence of PPARγ specific GW9662, in IR-3T3-L1 adipocytes. **(A)** *Pparγ*; **(B)** *Adpn* ; **(C)** *Glut4*; **(D)** *Scd1* ; **(E)** *Acc1*; **(F)** *Cd36*. All values are expressed as the mean ± SEM, n = 5 per group. ^*^P < 0.05, ^**^P < 0.01, and ^***^P < 0.001 when compared with nontreated insulin resistance (IR) group. ^#^*P* < 0.05, ^##^*P* < 0.01, and ^###^*P* < 0.001 compared with the group without specific antagonist GW9662 as marked in the figure.

**Figure 8:**
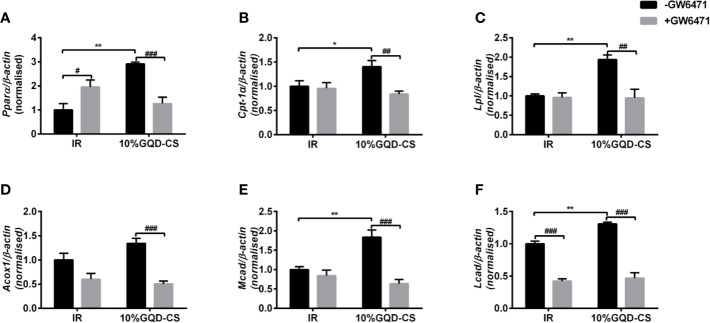
The mRNA expression level of glucolipid metabolism genes in presence of PPARα specific antagonist GW6471 in IR-3T3-L1 adipocytes. **(A)** *Pparα*; **(B)** *Cpt-1α* ; **(C)** *Lpl*; **(D)** *Acox1*; **(E)** *Mcad*; **(F)** *Lcad*. All values are expressed as the mean ± SEM, n = 5 per group. ^*^P < 0.05 and ^**^P < 0.01 when compared with non-treated IR group. ^#^*P* < 0.05, and ^##^*P* < 0.01, compared with the IR-3T3-L1 group without specific antagonist GW6471 as marked in the figure.

GQD-CS activated transcriptions of lipid metabolic genes including *Ppar/α*, *Pgc-1α*, *Cebpα*, *Cpt-1α*, *Lpl*, *Acc1*, *Cd36*, *Fasn*, *Lcad*, *Mcad*, *Acox1*, *Glut1*, *Pepck*, and *Gk*. Moreover, GQD-CS decreased transcriptions of lipid metabolic genes *Srebp-1c* and *Fabp4* (**Figure 6**). Specific PPARγ antagonist GW9662 significantly inhibited the mRNA expression of *Pparγ* and its downstream target genes *Adpn* and *Glut4* in 10% GQD-CS treated group and rosiglitazone group in IR-3T3-L1 adipocytes (**Figure 7**). In addition, 10% GQD-CS treated group did not significantly increase *Scd1* mRNA expression, partly because SCD1 is transcriptionally regulated by series of regulators in addition to PPAR-related signals. GW6471 inhibited mRNA expression of *Ppara* and its target genes *Cpt-1α, Lpl, Mcad, Lcad*, and *Acox1* in 10% GQD-CS treated group (**Figure 8**).

### GQD-CS Activated PPARγ and PPARα Signaling Systems by Multiple Pathways to Regulate Glucose and Lipid Gene Expression

**Figure 9:**
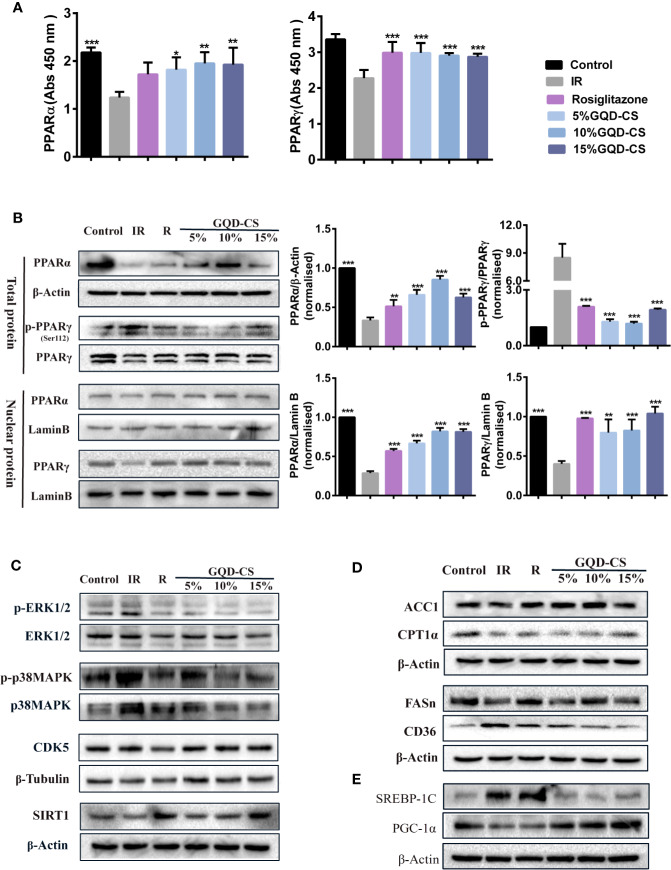
The effect of serum (GQD-CS) with PPARγ/PPARα activation in the IR-3T3-L1 adipocytes. **(A)** PPARα/PPARγ protein binding activities with target peroxisome proliferator response element (PPRE) element, n=5 per group. **(B)** Total PPARα/PPARγ protein levels and nuclear PPARα/PPARγ protein levels. **(C)** PPARγ/α upstream phosphorylated kinase expression including p-p38MAPK, p-ERK1/2, CDK5, and deacetylase SIRT1 expression. **(D)** PPARγ/α downstream target protein. **(E)** Other nuclear transcription factors crosstalk with PPARγ/α. In all WB experiments, data showed representing per group of n = 3, all values are expressed as the mean ± SEM. ^*^P < 0.05, ^**^P < 0.01, and ^***^P < 0.001 when compared with IR-3T3-L1 group. The target protein expression levels were normalized to that of β-actin or β-tubulin expression levels. The nuclear protein expression levels were normalized to that of Lamin B expression level.

Compared with IR group, GQD-CS treated groups (5%, 10%, and 15%) showed significantly increase in PPARγ and PPARα binding activities with perixosome proliferation-activated response elements of target genes (**Figure 9A**). Moreover, PPARγ and PPARα were elevated in total protein and nuclear protein samples of GQD-CS treated group (**Figure 9B**). Rosiglitazone treatments only increased PPARγ binding activity rather than PPARα binding activity. Moreover, GQD decreased upstream phosphorylation level of ERK1/2 and CDK5 leading whereas increased upstream phosphorylation level of p38MAPK and SIRT1 expression, which affect PPARγ and PPARα activity (**Figure 9C**). GQD and rosiglitazone treatments increased expression of lipid metabolism genes, including PGC-1α, ACC1, and FASn, but only GQD treatment decreased expression of SREBP-1C (**Figures 9D, E**).

## Discussion

Some traditional remedies and empirically selected drugs such as traditional Chinese Medicine (TCM) therapy have been proven to be beneficial and safe for clinical treatment, especially for chronic disease with long-time therapy (Leonti and Casu, 2013). Clinical research showed that many herbal medicines effectively improve IR through some promising target genes such as PPARs, GLUTs, PI3K, AMPK, and MAPKs involved in the insulin signaling pathway (Li et al., 2019). Given the clinical significance of GQD in treating T2DM, in-depth study on the molecular mechanism of GQD in regulating glucose and lipid homeostasis to improve IR may provide theoretical basis and treatment guidance. Here, GQD made not only good glycemic and body weight control but also improved blood lipid profiles with reduced TG, TC, and LDL-C in diabetic SD rats. Results are consistent with the clinical observation with long-term GQD and main active component berberine administration in DM patients (Lan et al., 2015; Tian et al., 2016). Dysfuction of WAT and imbalance of circulating metabolites including glucoses and lipids affect insulin signaling to modulate other peripheral tissue such as liver and muscle tissue (Rodent and Shulman, 2019). Our preliminary study showed that PPAR*γ* was activated to not only increase insulin-sensitive gene expression such as ADPN and GLUT4 but also promote TG-synthetic gene expression such as ACC1/2 and *FASn*, making fatty acids from excessive glucose in WAT of the GQD-treated diabetic rats (Luo et al., 2017). Except for our previous results, this study further showed that GQD treatment of diabetic rats experienced an increase in gene expression of *Pparα*, *Lcad*, *Mcad*, *Acox1*, *Lpl*, and *Sirt1* and an decrease in gene expression of *Srebp-1c*, *Gfat*, *Spot14*, and *Gadph*, which affected multiple pathways involved in lipid and glucose metabolism. Additionally, GQD concomitantly increased adipocytic PPARγ and PPARα expression and activity in WAT of diabetic rats, as a novel concomitant PPARα and PPARγ stimulator.

Notably, PPARα and PPARγ play a complementary role in the fine-tuning of lipid transport, catabolism and storage. PPARγ is the only highly expressed nuclear transcription factor in WAT, which activates GLUT4 and ADPN expression to improve insulin-stimulated glucose uptake and utilization to diminish high fat diet-induced IR (Moraes-Vieira et al., 2016; Gross et al., 2017; Han et al., 2017). Unexpectedly, GQD also stimulated PPARα expression and activity in WAT of diabetic rats, a tissue with a low capacity for fatty acid oxidation (Gross et al., 2017; Kersten and Stienstra, 2017). Activated PPARα increased *Lpl* expression to decompose TG into fatty acids, and elevated *Cpt-1α*, *Mcad*, *Lcad*, and *Acox1* expression to accelerate fatty acid oxidation. The mRNA expression of SIRT1 was decreased in WAT of diabetic SD rats and IR-3T3-L1 adipocytes, it is consistent that reduced expression of SIRT1 in human obesity may foster visceral adipose tissue expansion (Perrini et al., 2020). GQD also increased the expression of histone deacetylase SIRT1, which may inhibit adipocyte differentiation including PPARγ adipogenesis activity and initiate a broad program of mitochondrial gene expression and thermogenesis (Tang, 2016). PPARγ and PPARα appear to be closely interconnected, and activation of both may better improve glucose homeostasis and lipid metabolism to compose the anti-diabetic effects of GQD without weight gain.

To date, only two dual PPARα/γ dual agonists, lobeglitazone and saroglitazar, with predominant PPARα and moderate PPARγ activity with desired potency for the balance of PPARα vs PPARγ activations, have successfully applied in the clinical treatment of T2DM with dyslipidemia, which showed better efficacy and toxicity than TZDs as anti-diabetic drugs (Hong et al., 2018). In this study, the results indicated that GQD-CS increased GC with a time-dependent manner in IR-3T3-L1 adipocytes. The increase in secreted ADPN enhances insulin sensitivity associated with a shift toward smaller, more insulin-sensitive adipocytes following GQD-CS administration (Mclaugulin et al., 2007; Matsukawa et al., 2015), it is consistent with our previous TG-lowering results *in vitro* (Luo et al., 2017). In addition, more PPARα and PPARγ were shuttled into nuclear compartment to bind specific DNA promoter sequence, peroxisome proliferator response element (PPRE), which stimulated the expression of target genes involved in glucose and lipid metabolism in IR 3T3-L1 adipocytes under GQD treatment. Administration of specific PPARγ/α antagonists GW9662/GW6471 confirmed that GQD activated PPARγ and PPARα to change the transcription level of *Adpn*, *Glut4*, *Cpt-1α*, *Acox1*, *Mcad*, *Lcad*, *ApoaI*, *Lpl*, etc. The results indicated critical roles of adipocyte PPARα and PPARγ in the regulation of glucose and lipid metabolism.

**Figure 10:**
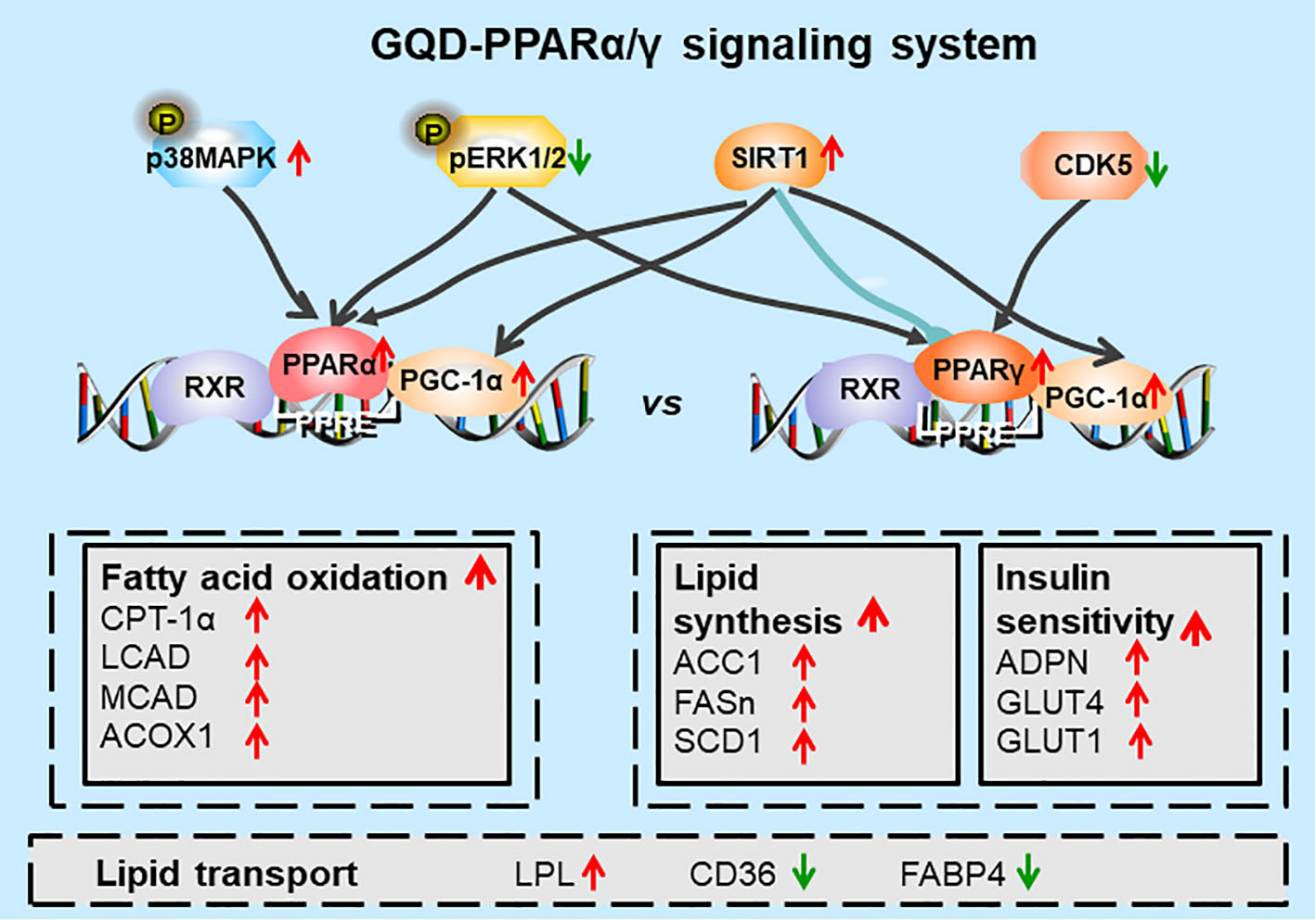
Diagram of predicted working model of Gegen Qinlian Decoction (GQD) as a novel PPARγ and PPARα agonist. GQD significantly concomitantly activated adipocytic PPARγ and PPARα, which undergo differentiate posttranslational modification, and bound to the specific promoter element peroxisome proliferator response element (PPRE). Activated PPARγ transcriptionally elevated ADPN and GLUT4 to increase insulin sensitivity to improve glucose metabolism, but activated lipogenesis genes such as ACC, FASn, and SCD1 that increase triglyceride (TG) content. Activated PPARα increased LPL expression to decompose TG into fatty acids whereas upregulated CPT-1α, LCAD, MCAD, and ACOX1 expression to accelerate fatty acid oxidation, thus, finally decreased TG accumulation for an encouraging lipid profile (Dubois et al., 2017). In sum, PPARγ and PPARα appear to be closely interconnected, which potentially provided cross-ordination between glucose homeostasis and lipid metabolism.

Additionally, GQD decreased the phosphorylation level of ERK1/2 and CDK5 to elevate PPARγ and PPARα activities in IR-3T3-L1 adipocytes. Increases in phosphorylation level of p38MAPK and SIRT1 expression may be attributed to inhibit PPARγ adipogenesis activity and activate PPARα activity through differentiated recruitment of cofactors including PGC-1α for mitochondrial energy homeostasis (**Figure 9C**) (Barger et al., 2001; Kim et al., 2013; Banks et al., 2015; Brunmeir and Xu, 2018). In this study, PGC-1α expression was elevated in WAT of diabetic rats or IR-3T3-L1 with GQD treatment, which it may be partially through deacetylation of PGC-1α by SIRT and phophorylation of PGC-1α to increase its protein stability for co-activation of PPARα and/or PPARγ transcription (Miller et al., 2019). As the combined results, GQD intervened a diverse array of PPARγ/α upstream kinase signaling transduction cascades to avoid excessive PPARγ adipogenesis. Compared with the rosiglitazone-treated group, GQD elevated *Cpt-1α* expression, decreased diabetic biomarker *Fabp4* expression and lipid-sensor regulator SREBP-1C expression, which produced an encouraging lipid profile with TG decrease in IR-3T3-L1 adipocytes. These results suggested that GQD intervention is through a novel, non-TZD PPARγ and PPARα dual signals to cause antihyperglycemic and TG-lowering effects, which jointly optimized the expression of target gene profiles to promote fatty acid oxidation and accelerate glucose uptake and utilization for more balanced glucose and lipid metabolism than PPARγ full agonist rosiglitazone without stimulating PPARα activity (**Figure 10**). PPARγ and PPARα are opposite regulators of TG decomposition and synthesis, thus largely affect adipose lipid turnover (Arner et al., 2019). Thus, the metabolic effects on dynamic balance of lipid profiles in GQD treatment should be further investigated to establish clinic usage of GQD including diabetes, dyslipidemia, and obesity.

## Conclusions

GQD has anti-diabetic and anti-IR effects in diabetic rats and IR-3T3-L1 adipocytes, partially through intervening a diverse array of PPARγ and PPARα upstream and downstream signaling transduction cascades, which jointly optimized the expression of target gene profiles to promote fatty acid oxidation and accelerate glucose uptake and utilization to maintain a normal glucose homeostasis. This study provides a new insight into the molecular mechanisms of anti-diabetic action of GQD as a PPARγ and PPARα dual agonist to accelerate the clinical use.

## Data Availability Statement

All datasets generated for this study are included in the article/**Supplementary Material**.

## Ethics Statement

The animal study was reviewed and approved by the Animal Ethics Committee of Jiangxi University of Traditional Chinese Medicine.

## Author Contributions

JT, GX, and CC designed experiments, JT, SZ, BL, XL, LJ, and XY performed experiments and analyzed the data. JT drafted the manuscript. CC and RZ contributed to scientific discussion and manuscript finalization.

## Funding

This work was funded by the National Nature Science Foundation of China (grant number 81460621 and 81960809), Jiangxi Nature Science Foundation (grant number 20143ACB20010 and 20171BAB205094), Science and Technology Planning Project of Jiangxi Education Department (grant number GJJ180665), and TCM project of Jiangxi University of Traditional Chinese Medicine (grant number JXSYLXK-ZHYAO121).

## Conflict of Interest

The authors declare that the research was conducted in the absence of any commercial or financial relationships that could be construed as a potential conflict of interest.

## Abbreviations

ACC1/2, acetyl-CoA carboxylase alpha/beta; ACOX1, acyl-CoA oxidase 1; ADPN, adiponectin, C1Q and collagen domain containing; CD36, fatty acid translocase; CDK5, cyclin-dependent kinase 5; CPT-1α, carnitine palmitoyl-transferase 1α; DEX, dexamethasone; DM, diabetes mellitus; ERK1/2, extracellular signal-regulated kinase1/2; FABP4, fatty acid binding protein 4; FASn, fatty acid synthase; FBG, fasting blood glucose; GAPDH, glyceraldehyde-3-phosphate dehydrogenase; GFAT, glutamine–fructose-6-phosphate transaminase 1; GLUT2/4 (SLC2A2/4), glucose transport protein 2/4; GQD, Gegen Qinlian Decoction; HDLC, high-density lipoprotein cholesterol; IBMX, 3-isobutyl-1-methylxanthine; IR, insulin resistance; HFD, high-fat diet; HPLC, high performance liquid chromatography; LCAD (ACADL), acyl-CoA dehydrogenase long chain; LDL-C, low density lipoprotein cholesterol; LPL, lipoprotein lipase; MCAD (ACADM), acylCoA dehydrogenase medium chain; p38MAPK, p38 mitogen-activated protein kinases; PPARα/γ, peroxisome proliferators-activated receptor α/γ; PGC-1α (PPARγC1α), peroxisome proliferators-activated receptor γ coactivator-1α; qPCR, quantitative polymerase chain reaction; SCD1, stearoyl-CoA desaturase 1; SD, Sprague-Dawley; SIRT1, silence information regulator 1, Spot14 (THRSP), thyroid hormone responsive; STZ, streptozotocin; T2DM, type 2 diabetes mellitus; TC, total cholesterol; TCM, traditional Chinese medicine; TG, triglyceride; TNFα, tumor necrosis factor α; TZDs, thiazolidinediones; UPLC-MS/MS, performance liquid chromatography tandem mass spectrometry.
