# Supplementary material for: Gegen Qinlian Decoction Coordinately Regulates PPARγ and PPARα to Improve Glucose and Lipid Homeostasis in Diabetic Rats and Insulin Resistance 3T3-L1 Adipocytes
Source: Front Pharmacol. 2020 Jun 11;11:811. doi: 10.3389/fphar.2020.00811 (PMC7300300; doi:10.3389/fphar.2020.00811)
Supplement: Supplementary file 1 [file Image_1.pdf]

Supplementary files:

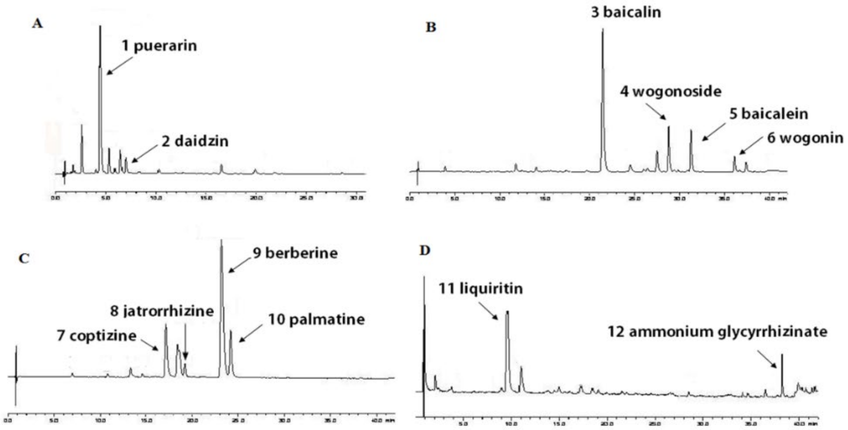

Figure S1. HPLC fingerprints of each single herb, including the chemical identification of the main marker compounds. (A) HPLC-UV chromatogram of Ge-Gen detected at 250 nm, (B) HPLC-UV chromatogram of Huang-Qin detected at 275 nm, (C) HPLC-UV chromatogram of Huang-Lian detected at 345 nm and (D) HPLC-UV chromatogram of Gan-Cao detected at 272 nm.
