# Supplementary material for: Gegen Qinlian Decoction Coordinately Regulates PPARγ and PPARα to Improve Glucose and Lipid Homeostasis in Diabetic Rats and Insulin Resistance 3T3-L1 Adipocytes
Source: Front Pharmacol. 2020 Jun 11;11:811. doi: 10.3389/fphar.2020.00811 (PMC7300300; doi:10.3389/fphar.2020.00811)
Supplement: Supplementary file 2 [file Table_1.docx]

**Supplementary files:**

Table S1. Plant full scientific name.

| **No** | **Plant common name** | **Plant full scientific name MANDATORY DATABASE Kew MPNS** | **Plant full scientific name Alternative Database (this column is optional)** |
| --- | --- | --- | --- |
| 1 | Gegen | Pueraria lobata (Willd.) Ohwi | Puerariae Lobatae Radix |
| 2 | Huanglian | Coptis chinensis Franch. | Coptidis Rhizoma |
| 3 | Huangqin | Scutellaria baicalensis Georgi | Scutellariae Baicalensis Radix |
| 4 | Zhigancao | Glycyrrhiza uralensis Fisch. | Glycyrrhizae Radix et Rhizoma Praeparata cum Melle |
