# Supplementary material for: Gegen Qinlian Decoction Coordinately Regulates PPARγ and PPARα to Improve Glucose and Lipid Homeostasis in Diabetic Rats and Insulin Resistance 3T3-L1 Adipocytes
Source: Front Pharmacol. 2020 Jun 11;11:811. doi: 10.3389/fphar.2020.00811 (PMC7300300; doi:10.3389/fphar.2020.00811)
Supplement: Supplementary file 3 [file Table_2.docx]

**Supplementary files:**

Table S2. qPCR primers for rat adipose tissue.

| GeneBank | Symbol | Forward Sequence  (5’-3’) | Reverse Sequence  (5’-3’) | Tm  (℃) | Product  length |
| --- | --- | --- | --- | --- | --- |
| NM_031144 | β-Actin | CCACCATGTACCCAGGCATT | AGGGTGTAAAACGCAGCTCA | 58~63 | 253bp |
| NM_017340 | ACOX1 | TGTCTGTCACTTCTGTCGCC | AGGCCAACAGGTTCCACAAA | 60 | 359bp |
| NM_012524 | CEBPα | CCATCCGCCTTGTGTGTACT | TAGACGCGCACACTGACATT | 60 | 192bp |
| NM_001005879.1 | GFAT1 | GCGCCTTTGCTCTTGTGTTT | TCTTGTCTTTGCCTGTTCTGTA | 60 | 150bp |
| NM_017008.4 | GAPDH | GCATGGCCTTCCGTGTTCC | GGGTGGTCCAGGGTTTCTTACTC | 60 | 337bp |
| NM_012819 | LCAD | TCCGCCCGATGTTCTCATTC | AGGGCCTGTGCAATTTGAGT | 60 | 327bp |
| [NM_012598](http://www.ncbi.nlm.nih.gov/entrez/viewer.fcgi?db=nucleotide&id=148747493) | LPL | ACTCCTACTTCCGCTGGTCA | GGTTTGTCCAGTGTCAGCCC | 60 | 204bp |
| NM_016986.2 | MCAD | CAAGAGAGCCTGGGAACTTG | CCCCAAAGAATTTGCTTCAA | 60 | 154bp |
| NM_013196 | PPARα | GGCTCTGAACATTGGCGTTC | CAAGGGGACAACCAGAGGAC | 60 | 96bp |
| NM_001145366.1 | PPARγ | CCTGGACCTCTGCTGGTGAT | GCTGGAGAAATCAACCGTGG | 60 | 265bp |
| NM_031347 | PGC-1α | GACATAGAGTGTGCTGCCCTG | ACCGCTAGCAAGTTTGCCTC | 63 | 227bp |
| NM_139192 | SCD1 | CGTGGCTTCTTGTGGTGTTG | ATACCAGGAGGAATGGGCCT | 60 | 243bp |
| [XM_017588053](https://www.ncbi.nlm.nih.gov/entrez/viewer.fcgi?db=nucleotide&id=1046829004) | SIRT1 | GGCAGACAATTTAATGGGGTGAA | GAAGTCCACAGCAAGGCGAG | 58 | 115bp |
| [NM_012703](https://www.ncbi.nlm.nih.gov/entrez/viewer.fcgi?db=nucleotide&id=58219501) | Spot14 | CTGAGCGCATCTGTGGACTT | GGCCGCTTGCTATTACCTCT | 58 | 139bp |
| NM_001276708 | SREBP-1C | GGAGCCATGGATTGCACATT | AGGAAGGCTTCCAGAGAGGA | 63 | 191bp |
