# Supplementary material for: Gegen Qinlian Decoction Coordinately Regulates PPARγ and PPARα to Improve Glucose and Lipid Homeostasis in Diabetic Rats and Insulin Resistance 3T3-L1 Adipocytes
Source: Front Pharmacol. 2020 Jun 11;11:811. doi: 10.3389/fphar.2020.00811 (PMC7300300; doi:10.3389/fphar.2020.00811)
Supplement: Supplementary file 4 [file Table_3.docx]

**Supplementary files:**

Table S3. qPCR primers for 3T3-L1 adipocytes.

| **GeneBank** | **Symbol** | **Forward Sequence**  **(5’-3’)** | **Reverse Sequence**  **(5’-3’)** | **Tm**  **(℃)** | **Product**  **length** |
| --- | --- | --- | --- | --- | --- |
| NM_007393.5 | β-Actin | CCACCATGTACCCAGGCATT | AGGGTGTAAAACGCAGCTCA | 58-63 | 253bp |
| [NM_015729.3](https://www.ncbi.nlm.nih.gov/entrez/viewer.fcgi?db=nucleotide&id=429484482) | ACOX1 | GGAATCTGGAGATCACGGGC | GGCATGTAACCCGTAGCACT | 60 | 230bp |
| [NM_009605.4](http://www.ncbi.nlm.nih.gov/entrez/viewer.fcgi?db=nucleotide&id=87252710) | ADPN | AGCCGCTTATGTGTATCGCT | GAGTCCCGGAATGTTGCAGT | 60 | 154bp |
| [NM_133360.2](file:///\\www.ncbi.nlm.nih.gov\entrez\viewer.fcgi?db=nucleotide&id=125656172) | ACC1 | ATGCGATCTATCCGTCGGTG | AGCAGTTCTGGGAGTTTCGG | 60 | 266bp |
| [L23108.1](file:///\\www.ncbi.nlm.nih.gov\entrez\viewer.fcgi?db=nucleotide&id=567198) | CD36 | TGTGGAGCAACTGGTGGATG | CGCCAACTCCCAGGTACAAT | 60 | 179bp |
| NM_031559 | CPT-1α | CCTACCACGGCTGGATGTTT | TACAACATGGGCTTCCGACC | 60 | 103bp |
| [NM_007988.3](http://www.ncbi.nlm.nih.gov/entrez/viewer.fcgi?db=nucleotide&id=93102408) | FASn | GGGTGTGAGTGGTTCAGAGG | ACAGAGGTGTTCGGCTTCAG | 63 | 267bp |
| [NM_009204.2](file:///\\www.ncbi.nlm.nih.gov\entrez\viewer.fcgi?db=nucleotide&id=118026924) | GLUT4 | AAACCCAAGGGCTGCTGTAT | AAGTGCAAAGGGTGAGTGAGG | 60 | 133bp |
| [NM_011400.3](https://www.ncbi.nlm.nih.gov/entrez/viewer.fcgi?db=nucleotide&id=165377225) | GLUT1 | CGATCTGAGCTACGGGGTCT | ACGGACGCGCTGTAACTATG | 60 | 76bp |
| [NM_007381.4](https://www.ncbi.nlm.nih.gov/entrez/viewer.fcgi?db=nucleotide&id=425876784) | LCAD | GTCCGATTGCCAGCTAATGC | CACAGGCAGAAATCGCCAAC | 60 | 115bp |
| NM_008509.2 | LPL | AAACCCCAGCAAGGCATACA | ATTTGTGGAAACCTCGGGCA | 60 | 310bp |
| [NM_007382.5](https://www.ncbi.nlm.nih.gov/entrez/viewer.fcgi?db=nucleotide&id=425854813) | MCAD | AAAAGAGCCTGGGAACTCGG | CCATACGCCAACTCTTCGGT | 60 | 110bp |
| [NM_001113418.1](https://www.ncbi.nlm.nih.gov/entrez/viewer.fcgi?db=nucleotide&id=164663879) | PPARα | AACTGACGTTTGTGGCTGGT | GCTCTCTGTGTCCACCATGT | 60 | 108bp |
| NM_011146.3 | PPARγ | GCTGTTATGGGTGAAACTCTGG | ATAGGCAGTGCATCAGCGAA | 60 | 74bp |
| [NM_008904.2](file:///\\www.ncbi.nlm.nih.gov\entrez\viewer.fcgi?db=nucleotide&id=238018130) | PGC-1α | AGTTCACTCTCAGTAAGGGGC | CCAACCAGAGCAGCACACT | 63 | 166bp |
| [NM_009127.4](http://www.ncbi.nlm.nih.gov/entrez/viewer.fcgi?db=nucleotide&id=227908811) | SCD1 | CGATAAAAGGGGGCTGAGGAA | TGACTCTCGGGATGGGTGTT | 60 | 124bp |
