# Supplementary material for: Gegen Qinlian Decoction Coordinately Regulates PPARγ and PPARα to Improve Glucose and Lipid Homeostasis in Diabetic Rats and Insulin Resistance 3T3-L1 Adipocytes
Source: Front Pharmacol. 2020 Jun 11;11:811. doi: 10.3389/fphar.2020.00811 (PMC7300300; doi:10.3389/fphar.2020.00811)
Supplement: Supplementary file 5 [file Table_4.docx]

**Supplementary files:**

Table S4. Significant change of adipocytic genes for IPA pathway analysis.

| **Gene Symbol** | **Entrez Gene Name** | **Fold Change** | **p-value** | **Type(s)** |
| --- | --- | --- | --- | --- |
| ACACA | acetyl-CoA carboxylase alpha | 2.822 | 0.0038 | enzyme |
| ACACB | acetyl-CoA carboxylase beta | 4.251 | 0.016 | enzyme |
| ACADL | acyl-CoA dehydrogenase long chain | 2.02 | 0.0011 | enzyme |
| ACADM | acyl-CoA dehydrogenase medium chain | 2.119 | 0.0002 | enzyme |
| ACOX1 | acyl-CoA oxidase 1 | 1.895 | 0.0009 | enzyme |
| ADIPOQ | adiponectin, C1Q and collagen domain containing | 1.903 | 0.0155 | other |
| CEBPA | CCAAT enhancer binding protein alpha | 6.364 | 0.0013 | transcription regulator |
| GAPDH | glyceraldehyde-3-phosphate dehydrogenase | -1.659 | 0.0048 | enzyme |
| GFPT1 | glutamine--fructose-6-phosphate transaminase 1 | -3.89 | 0.0001 | enzyme |
| LPL | lipoprotein lipase | 3.639 | 0.0027 | enzyme |
| OLR1 | oxidized low density lipoprotein receptor 1 | 6.132 | 0.0001 | receptor |
| PPARA | peroxisome proliferator activated receptor alpha | 1.795 | 0.00111 | nuclear receptor |
| PPARG | peroxisome proliferator activated receptor gamma | 2.692 | 0.019 | nuclear receptor |
| PPARGC1A | coactivator 1 alpha | 1.721 | 0.19 | transcription regulator |
| SIRT1 | silence information regulator 1 | 2.81 | 0.0097 | transcription regulator |
| SLC2A2 | solute carrier family 2 member 2 | 2.102 | 0.004 | transporter |
| SLC2A4 | solute carrier family 2 member 4 | 1.947 | 0.0115 | transporter |
| SREBF1 | sterol regulatory element binding transcription factor 1 | -2.357 | 0.005 | transcription regulator |
| THRSP | thyroid hormone responsive | -2.382 | 0.0032 | other |

Notes: ACADL (LCAD); ACADM (MCAD); ADIPOQ (ADPN); SLC2A2 (GLUT2); SLC2A4 (GLUT4); SREBF (SREBP); THRSP (Spot14).
